# New-Generation Sequencing Technology in Diagnosis of Fungal Plant Pathogens: A Dream Comes True?

**DOI:** 10.3390/jof8070737

**Published:** 2022-07-16

**Authors:** Maria Aragona, Anita Haegi, Maria Teresa Valente, Luca Riccioni, Laura Orzali, Salvatore Vitale, Laura Luongo, Alessandro Infantino

**Affiliations:** Council for Agricultural Research and Economics (CREA), Research Centre for Plant Protection and Certification (CREA-DC), Via C.G. Bertero 22, 00156 Rome, Italy; anita.haegi@crea.gov.it (A.H.); mariateresa.valente@crea.gov.it (M.T.V.); luca.riccioni@crea.gov.it (L.R.); laura.orzali@crea.gov.it (L.O.); salvatore.vitale@crea.gov.it (S.V.); laura.luongo@crea.gov.it (L.L.); alessandro.infantino@crea.gov.it (A.I.)

**Keywords:** metabarcoding, metagenomics, fungal taxonomy, plant disease diagnosis, filamentous fungal pathogens, high-throughput sequencing

## Abstract

The fast and continued progress of high-throughput sequencing (HTS) and the drastic reduction of its costs have boosted new and unpredictable developments in the field of plant pathology. The cost of whole-genome sequencing, which, until few years ago, was prohibitive for many projects, is now so affordable that a new branch, phylogenomics, is being developed. Fungal taxonomy is being deeply influenced by genome comparison, too. It is now easier to discover new genes as potential targets for an accurate diagnosis of new or emerging pathogens, notably those of quarantine concern. Similarly, with the development of metabarcoding and metagenomics techniques, it is now possible to unravel complex diseases or answer crucial questions, such as “What’s in my soil?”, to a good approximation, including fungi, bacteria, nematodes, etc. The new technologies allow to redraw the approach for disease control strategies considering the pathogens within their environment and deciphering the complex interactions between microorganisms and the cultivated crops. This kind of analysis usually generates big data that need sophisticated bioinformatic tools (machine learning, artificial intelligence) for their management. Herein, examples of the use of new technologies for research in fungal diversity and diagnosis of some fungal pathogens are reported.

## 1. Introduction

Plant pathologists are aware that a disease epidemic, the so-called “square pyramid disease”, is under the control of many factors, namely plant, pathogen, environment (microbiome), time, and human social systems, considered as the drivers of disease development [1,2]. This is particularly true for soilborne diseases, considering the huge amount of microorganisms per g of soil (about 10^29^) [3] and the vast and still unknown estimated fraction of uncharacterized fungal species [4,5,6]. The definition of the species involved in a particular disease is a key factor to be considered when studying the epidemiology of the disease and to guarantee the success of any disease-control strategy (genetical, chemical, biological). The fast and accurate diagnosis of fungal and oomycete pathogens, which cause severe losses in agriculture and forestry, is urgent for the reduction of the use of chemical products and yield losses [7]. Diagnostic methods based on pathogen culturing and direct morphology observation are time-consuming and often not reliable, such as those for non-culturable pathogens. Molecular methods using specific oligonucleotide primers or probes, such as PCR-based protocols, DNA hybridization-based techniques, and DNA sequence analysis, are more accurate but sometimes yield false-positive results and/or are unable to detect unknown species or all the species present in a complex community [8]. DNA analysis of specific marker genes, such as ITS and TEF for fungi, obtained by Sanger sequencing of PCR products, takes several days but possesses high resolution at the taxonomic level except when polymorphic alleles are amplified, which is common in noncoding regions of eukaryotes. The advent of next-generation sequencing technologies (NGS) has allowed the possibility to quickly sequence thousands of microorganisms in a massively parallel approach, with decreasing costs and improved precision. Monitoring of diseases of complex etiology or changes of microbial populations in different environments (marine, soil, air) is now possible by rapidly collecting and analyzing environmental DNA samples (eDNA) without the need of taxonomic expertise, at costs even more affordable. The conventional techniques for the isolation of the pathogen from symptomatic tissue or from soil, followed by the morphological identification, have been joined (and often overcome) by the availability of DNA-based tools that improved our ability to rapidly and cheaply diagnose the emergence and spread of a particular pathogen and to quantify its biomass into the plant or into the soil [9,10,11,12]. Nevertheless, these approaches are time-consuming. Techniques applied for the molecular diagnosis of pathogens are limited by the previous knowledge of the disease agent, thus rendering difficult the possibility to identify new or emerging pathogens of concern, mostly in the climate-change scenarios [13]. However, they are continuously and rapidly evolving, and it is difficult to foresee which one will dominate the market in the near future [14,15,16]. The use of high-throughput DNA sequencing (HTS) technologies has revolutionized studies in plant and soil biology, allowing a more realistic assessment of biodiversity in terrestrial communities [17]. A range of HTS platforms exists, of the so-called second and third generations, and the introduction on the market of recent new models of sequencers, such as Illumina’s MiSeq, the Ion Personal Genome Machine (PGM) (Life Technologies), PacBio’s Sequel IIe, and Oxford Nanopore Technologies’s MinION system, is revolutionizing this sector. In addition, more and more pipelines for data analysis are under development, contributing to the appearance of several recent articles dealing with application of HTS technologies in diagnosis of filamentous fungal plant pathogens up to species-level resolution. In this review, we provide an overview of omics techniques, and we try to validate the thesis that the use of HTS technologies in taxonomy of fungal pathogens is spreading and is more and more a reality and not just a dream anymore although several biases still need to be solved and are here analyzed.

Here, we review the use to date of HTS technologies in diagnosis of filamentous fungal pathogens. Comparison with the diagnosis of viruses and bacteria plant pathogens is mentioned, and we refer to other articles [14,16]. The technical principles and pros and cons of HTS technologies are also dealt with, and examples of their use for diagnosis of some fungal pathogens and analysis of genetic diversity are reported. We provide an overview of core experimental steps for metabarcoding and of its use in diagnosis of fungi and oomycetes, including plant and forest pathogens, and the use in assessment of fungal biodiversity in diverse environments (soil, water). Metagenomic and metatranscriptomic approaches are also described in their applications for diagnostic purposes. Then, we report the case of multilocus analysis (MLST) as an example of a methodology for which NGS technologies have made it possible to shorten the times of experimental analyses and those of data analysis as well as to reduce costs. Phylogenomics and genome-wide association studies (GWAS) are also treated because they represent a chance to better define at genome scale the basis of fungal speciation and to discover new effectors and/or pathogenic genetic determinants that act as putative specific markers in diagnosis. We focus our attention on how HTS technologies also deeply influence fungal taxonomy that, in some cases, has been redrawn, and a case study of a tomato fungal pathogen is reported. Then, we deal with the promising applications in diagnosis of fungal pathogens for phytosanitary purposes, including surveillance, certification of plant propagation material, monitoring of imported plant material, and more. In this field, the use of HTS methods is almost a challenge and still a dream for the detection of fungal pathogens, but the examples realized for viruses and bacteria are strongly encouraging. Throughout the review, we highlight the need to manage the large amount of data generated by the applications of HTS technologies and, as consequence, the availability of even more friendly bioinformatic tools (machine learning, artificial intelligence).

We complete the review with a synthesis of the economic and technical aspects to be considered before the huge possibilities offered by HTS technologies in the diagnostic field of fungal pathogens become a reality and applicable also at the public institution level for routine analyses.

## 2. High-Throughput DNA Sequencing (HTS) Technologies

High-throughput platforms for analysis of fungal communities currently include the second and third generation of HTS technologies (Box 1). HTS-based approaches enable in-depth characterization of the community composition of both prokaryotic (bacteria and archaea) and eukaryotic (protists, fungi, and microfauna) microorganisms. Especially in soil, a large fraction of microorganisms are uncultivable and can be detected only using molecular approaches [18].

There are two main approaches to estimate the identity and the relative species abundance in complex mixtures using HTS:

(1) Metabarcoding: PCR amplification of taxonomic marker genes (DNA barcode), followed by HTS and comparison to a DNA barcoding database;
(2) Metagenomics: the extracted DNA from the bulk sample is shotgun sequenced to analyze the collective genomes of the community.

Both methods have superior species detectability, require lower effort, cause no ecosystem disturbance, and allow detection without a priori knowledge of species compared to Sanger sequencing after PCR amplification of specific target genes [19].

Nevertheless, it is essential that the procedures are optimized and standardized. The lack of standardization makes it difficult to compare different studies. Moreover, difficulty in estimating DNA degradation rates could allow the misrepresentation of species presence. These approaches involve several processing steps, each of which might introduce significant biases that can considerably compromise the reliability of the metabarcode/metagenome output.

### Overview of Core Experimental Steps for Metabarcoding

The workflow of experimental procedures for metabarcoding approach is shown in Table 1.

The success of DNA metabarcoding mainly depends on the selection of the appropriate DNA marker gene (Table 1—Amplicon production). The chosen primers should have an appropriate coverage of the target group (i.e., taxonomic coverage), the efficient exclusion of outgroups (i.e., taxonomic specificity), and the ability to discriminate taxa based on nucleotide variability of the amplified marker (i.e., taxonomic resolution) [18]. It is possible to use customer-designed primers pairs, such as in the genome-enhanced detection and identification (GEDI) approach described below; nevertheless, it is also important to utilize universal primers on barcode to allow comparison between different studies. To characterize fungal communities in both DNA barcoding and metabarcoding analyses, the internal transcribed sequence (ITS) region of the rRNA is the most broadly used marker due to its multiple copy numbers and optimal species-level resolution in most groups [20], even if in some groups (for example *Trichoderma, Fusarium,* or *Oomycetes*), not all species are well-resolved with ITS region.

The core procedure of metabarcoding methodology is the high-throughput sequencing (HTS) that allows to sequence at the same time all the amplicons produced by PCR step, which is representative of all organisms present in the sample. Different sequencing platforms are now available and are rapidly improving (Box 1).

The sequencing data are then processed in several steps, including (i) demultiplexing of barcoded samples, (ii) pair-end (Illumina) assembly, (iii) removal of chimeric reads, (iv) quality filtering, (v) sequence clustering, and (vi) comparison of the representative sequences to a reference database.

The key points of metabarcoding sequencing data analysis are represented by (i) sequence clustering and (ii) taxonomic or (iii) functional assignment by comparison to databases.

Sequence reads are clustered based on their homology. In most metabarcoding works, reads sharing a predefined level of similarity (generally between 95% and 99%) are assembled into operational taxonomic units (OTUs). A level of 97% homology is normally used. To improve taxonomic resolution and reproducibility of identified taxa, amplicon sequence variant (ASV) approaches have been developed: it uses only unique, identical sequences for downstream community analyses corresponding to 100% OTUs similarity.

Once the reads are clustered, they must be assigned to a taxon (taxonomic assignments) or to a function (functional assignments) based on reference databases. The more curated and complete these databases are, the better is the information obtained. Identification of fungal and other eukaryote ITS sequences is usually performed on the UNITE reference data set (https://unite.ut.ee, accessed on 16 March 2022) because it is the largest curated database and includes multiple non-fungal reads to facilitate separation of fungi from other eukaryotes [21,22]. For functional assignments, the *FUNGuild* database [23] or *FungalTraits* database [24] can be used.

Different software pipelines are available to perform these processing steps aimed to analyze metabarcoding sequencing data, with QIME, MOTHUR, and DADA2 being the most used ones. There is no consensus concerning the most appropriate bioinformatic approach for the analysis of fungal metabarcoding data, and Pauvert et al. [25] highlighted the importance of carefully selecting the bioinformatic approach to be used according to the objective of the metabarcoding study.

It is worth to mention that NGS microbiome-based diagnostics generate a huge amount of data that makes the use of machine learning or other resources imperative for the assessment of human, plant, and soil health [26,27,28,29]. The ability of bioinformatic approaches to recover fungal strains and the relative abundances of the strains recovered varied greatly. Some sequence analysis tools (USEARCH and VSEARCH) detected almost all strains of the mock community but overestimated community richness, whereas others retrieved the actual richness and composition of the mock community more accurately (DADA2). The former two are more appropriate for the detection of target species, whereas the latter is more appropriate for community ecology studies [25].

Box 1 **Sequencing Platforms**

**Second-generation HTS methods—Short reads sequencing**

The development in the 2000s of next-, or second-, generation high-throughput sequencing (HTS) methods transformed molecular biology studies. In these sequencing platforms, which are able to address only short reads (<550 bases), DNA is sequenced by ligation or by synthesis. In the synthesis approach, used by most of the platforms, a polymerase is used, and a signal, such as a fluorophore or a change in ionic concentration, identifies the incorporation of a nucleotide into an elongating strand. DNA is blocked on a solid surface (solid-state, bead-based, and DNA nanoball generation), and clonal template populations are generated.

The approach of *454 pyrosequencing*, the first NGS instrument developed, is based on a strategy of “single-nucleotide addition”, which relies on a single signal to mark the incorporation of a dNTP into an elongating strand. As consequence, each of the four nucleotides must be added separately to a sequencing reaction to ensure only one dNTP is responsible for the signal. This platform is now obsolete.

* Illumina platform* uses, instead, terminator molecules (dNTPs) in which the ribose 3ʹ-OH group is blocked, thus preventing elongation. After the incorporation of a single dNTP to each elongating complementary strand, the surface is imaged to identify which dNTP was incorporated at each cluster. The fluorophore and blocking group can then be removed, and a new cycle can begin. Illumina dominates the short-read sequencing industry owed, in part, to its maturity as technology, a high level of cross-platform compatibility, and its wide range of platforms. It has a read length up to 300 bp and an overall accuracy rate of >99.5% [30].

The synthesis approach is also used in *Ion Torrent*, that is, the first NGS platform without optical sensing, where the signal is a change in ionic concentration. In *DNA nanoball sequencing*, DNA is instead sequenced by ligation.

Short-read sequencing platforms have the advantage to be mature sequencing technologies with high sequencing depth and low error rates, but they address only short reads (<550 bp); thus, only fragments of the genetic markers (barcode) are sequenced, resulting in the loss of taxonomic resolution—i.e., they do not always allow resolution to species level—and loss of phylogenetic information.

**Third-generation HTS methods—Long-reads sequencing**

In the 2010s, long-read platforms, such as PacBio single-molecule real-time (SMRT) sequencing (Pacific BioSciences Inc., California, USA, com) and nanopore sequencing (ONT, Oxford Nanopore Technologies Inc., Oxford, UK. https://nanoporetech.com, accessed on 10 July 2022), were introduced [31].

Currently, the most widely used long-read platform is the SMRT sequencing approach used by *PacBio.* The instrument uses a specialized flow cell with many thousands of individual picolitre wells—zero-mode waveguides (ZMW). The sequencing is based on DNA synthesis, with polymerase fixed to the bottom of the ZMW that synthetizes a single DNA molecule. This method had low sequencing depth (only hundreds rather than thousands of reads per sample), so SMRT platform developed a circular consensus sequence (CCS): a unique circular template repeatedly sequenced by the polymerase [32].

The MinION from *Oxford Nanopore Technologies*, developed in 2014, is the first user sequencer. This type of sequencer directly detects the DNA composition of a native ssDNA molecule: DNA is passed through a protein pore as current is passed through the pore, and this causes shifts in voltage that are characteristic of the DNA sequence in the pore, which can then be interpreted as a k-mer [33]. Recently, the company introduced a relevant improvement of this technique, named the rolling circle amplification (RCA) [34].

Initially, these long-read sequencers could not compete with short-read HTS platforms because of low sequencing depth and high error rates. Some of these limitations can be circumvented using unique molecular identifiers (UMIs) and proper bioinformatics tools [31]. Recently, long-read technologies have been improved, so they provide high-quality sequence data for up to 5 kb amplicons [31].

## 3. Metabarcoding

Metabarcoding has proven to be a cost-effective method to characterize microbial communities: it allows assessing sample biodiversity, providing deep taxonomic resolution, and comparing sample communities subjected to different treatments. From a bioinformatic point of view, it is easier to deal with than shotgun metagenomics (less storage space as well as less computational power) [35]. At present, it represents the most used molecular approach to characterize microbiota in environmental samples [18]. It offers new perspectives in the study of plant diseases of complex etiology, both in the aerial part and in the soil. Many examples of disease complexes of concern for agricultural crops, affecting both the aboveground and the underground tissues, are available: Fusarium Head Blight (FHB) is a serious disease of wheat in which many *Fusarium* species are involved, most of which are mycotoxin producers [36]. Illumina MiSeq using V3 Chemistry was used to define the wheat ear fungal community across a topographically heterogeneous environment [37]. Walder et al. [38], using PacBio CCS long reads sequencing, targeting a combination of the highly variable ITS and the D1–D2 -D3 segments of LSU region, tracked the changes of *Fusarium* spp. in crop residues following the use of different cover crops. Similarly in maize, the Illumina MiSeq amplification of bacterial 16S rRNA, fungal ITS and *Fusarium* spp. TEF1 regions shed light on the complex epidemiology of FHB by defining the presence and the co-occurrence of different phytopathogenic and beneficial microorganisms in maize stalks in rotation with wheat [39]. Illumina fungal and bacterial community profiles of wheat straws artificially inoculated with *Zymoseptoria tritici* allowed the understanding of the interactions and the dynamics between the pathogen and the whole microbial community over time [40]. Grapevine growing offers many positive applications of NGS techniques for the definition of the composition of microbial species relevant to winemaking [41,42]. The potential of NGS techniques has been utilized for the characterization of the grapevine trunk diseases clusters, namely *Eutypa*, Esca, *Botryosphaeria*, *Phomopsis* dieback, and Black foot, whose precise characterization is a prerequisite for the adoption of the most appropriate control strategies. Illumina short reads technology with optimized and universal primers targeting both ITS1 and ITS2 rDNA regions, confirmed the presence of the most representative species of each syndrome, but also allowed the discovery of species not-yet assigned to this complex [43,44]. As compared to diseases of wood, diagnosis of *Vitis* phylloplane diseases by NGS received less interest but it is foreseeable its use in the next future [45,46]. NGS was used to study the effect of elicitors or biocontrol agents on the leaf epiphytic populations [47,48]. Apple Replant Disease (ARD) is another serious disease of complex etiology affecting fruit trees, in particular apple and other Rosaceae replanted in the same site of previous cultivation [49,50]. Oomycetes (*Pythium* spp. and *Phytophthora* spp.) and fungi (*Cylindrocarpon* spp., *Rhizoctonia solani*) are the dominant species involved in many apple locations worldwide. The control of ARD is difficult due to the scarcity of registered chemicals and further complicated by the complex etiology of the disease. The use of NGS techniques highlighted significative differences in microbial composition among new and replant sites, mostly in beneficial bacteria populations (*Burkholderia* spp., *Microcoleus, Nocardioides*, sulfur-oxidizing bacteria and those involved in nitrogen-cycling) [51], and after green manure with *Brassica* spp. [52]. NGS has been further utilized to characterize the pathobiome of other crops, like oaks [53], ginseng [54] tomato [55,56], strawberry [57,58], potato [59], banana [60], ramie (*Boehmeria nivea*) [61]. Similarly, metabarcoding approach is widely used to study oomycetes, especially *Phytophthora* spp. (Box 2). The realistic assessment of biodiversity in fungal communities in diverse environments (soil, phylloplane, air, water) through the advent of HTS has boosted our ability to follow the fate of a particular pathogen within its changing environment, i.e., after a chemical or biological treatment, or when studying the effects of climate changes or agricultural practices on the development of a disease. The knowledge of the compositions and diversity of soil microbial communities is thus particularly needed for a better understanding of the structure and function of microbiota associated to different environments, i.e., roots, leaves, suppressive soils, degraded soils [62,63,64,65,66,67].

Several fungi can combine different lifestyles, i.e., pathogenic, saprophytic or symbiotic, so that their boundaries are often not clear. They can alter their lifestyle, such as in the adaptation of endophytes into parasites, and vice versa, by omics, tools new methods for exploring plant-microbe interaction and elucidating the different behaviors are developing [68]. Finally, microbiome studies are widely used in environmental studies, i.e., to define biodiversity status and implement conservation actions in protected areas [69,70], to define the role of several factors (host taxon and tissues, seasonality) in shaping the composition of both fungi and bacteria in tropical forests [71], or to compare the microbiomes of trees and of associated herbaceous plants used as phytoremediation [72].

Box 2 **Application of metabarcoding in *Phytophthora.***

Oomycetes are the causal agents of outbreaks especially in natural environments. Real-time qPCR remains the best method for oomycete diagnosis, however surveillance of *Phytophthora* genus in natural environments, as well as in urban forestry and nurseries, requires an untargeted screening methodology able to detect the different *Phytophthora* species: the untargeted NGS-based approaches, like metabarcoding or metagenomics approaches, appear as the most suitable technologies. Even if metagenomics has likely higher potential than metabarcoding, its application for *Phytophthora* spp. appears, now, too complicated due to the poor knowledge of *Phytophthora* genomes. Metabarcoding is rapidly gaining a great success for this genus, as for fungal pathogens. Pyrosequencing has been widely used to study *Phytophthora* communities in different environments [73,74,75,76,77]. In more recent years, 454 pyrosequencing became obsolete and even more *Phytophthora* metabarcoding studies are now performed using the most cost-effective Illumina platform [14]. Despite some current shortcomings, metabarcoding technology based on ITS1 region/Illumina platform showed to be a valuable tool for simultaneous detection and identification of *Phytophthora*/oomycetes in environmental samples and has been widely used to study these pathogens in plant material, soil, and water. This kind of approach has been used to study the diversity of soil-borne *Phytophthora* communities in natural ecosystems, like tropical forests in North French Guiana rainforest [78], declining holm oak forests [53], or anthropized ecosystems, like public gardens [79], and plant nurseries [80].

Metabarcoding showed that *Phytophthora* diseases in natural environments are emerging not only in association with casual introductions, or due to the proximity of wildlands to agricultural settings, but also, unexpectedly, in association with **restoration projects**. In fact, a gathering body of evidence is demonstrating the introduction and spread of invasive *Phytophthora* species in California wildlands as the result of restoration schemes involving planted native species raised in contaminated nurseries [81]. Based on this conclusion, different recent studies focus on the identification of *Phytophthora* species in forest nurseries plantings using metabarcoding to implement stringent biosecurity practices when raising stocks of native plants destined for planting onto ecologically sensitive sites [82,83].

The occurrence in the nurseries of *Phytophthora* spp., and more in general of plant pathogenic oomycetes, is not only a problem for forest ones but also for all other nurseries, especially those with international **trade of potted plants**. In these cases, plant pathogenic oomycetes could be rapidly transported over long distances not only by plants but especially within soil of potted plants, thus facilitating diseases outbreaks that threaten ecosystems, biodiversity and food security. For example, Rossman et al. [84] employed Illumina-based DNA metabarcoding to detect oomycetes in soil from internationally traded plants and showed the widespread presence of potentially plant pathogenic *Phytophthora* and *Pythium * species in internationally transported rhizosphere soils, with *Pythium * being the overall most abundant genus observed. The authors proposed the metabarcoding approach as a phytosanitary assessment tool for detection of plant pathogenic oomycetes.

At present, ITS1/Illumina-based approach is the most used for *Phytophthora* metabarcoding, even if ITS1 DNA barcode has poor taxonomic resolution to identify many oomycetes up to the species level [85]. Different works tried to change the DNA barcode region [86,87] but without success.

To balance the short reads/ITS1-based metabarcoding drawbacks, the molecular identification has been complemented with other approaches. Different Phytophthora’s community studies have complemented DNA metabarcoding with conventional leaf baiting isolation techniques. The major advantage of this combined approach is that baiting allows to obtain *Phytophthora* cultures useful for a better knowledge of isolates and Koch’s postulates validation. As expected, DNA metabarcoding revealed a higher number of *Phytophthora* taxa than baiting [85,88]. Sometimes, the identified taxa were not the same between the two methods [88], in other cases the two approaches revealed very similar *Phytophthora* communities [87].

Riddell et al. [89] utilized real-time PCR to detect specifically *Phytophthora austrocedri* within infected *Juniperus communis* woodland and the metabarcoding approach to investigate the wider diversity of *Phytophthora* species present in the same site. Metabarcoding identified DNA from a diverse range of *Phytophthora* species but the main target pathogen, *P. austrocedri*, was not amplified. Differently, Català et al. [76] identified different *Phytophthora* species by metabarcoding which revealed that an uncultured *Phytophthora* taxon was the predominant species in that area. Thus, they used metabarcoding data to set up a real-time PCR assay, for the detection of this uncultured *Phytophthora* taxon.

As for fungi, short-reads HTS drawbacks can be overcome by long reads sequencing approaches, like PacBio and Oxford Nanopore Technologies (ONT), improving taxonomic resolution of markers and barcodes. Abad et al. [90] reported that correct identification of *Phytophthora* to species levels in environmental DNA is possible targeting the full ITS rDNA via the miniaturized MinION portable device. Different studies on environmental fungal communities, that also includes *Oomycetes*, have been also performed with PacBio Sequencing [83,91], but no long-read sequencing method has been found to be applied specifically to *Phytophthora* genus.

## 4. Metagenomics and Metatranscriptomics

Over the past two decades, shotgun metagenomic studies have played an important role in the analysis of the taxonomic and functional profiles of microbial communities. NGS-based metagenomics technologies were first used for pathogen detection in clinical field [92] and are now more and more used in plant disease diagnosis [16]. Shotgun metagenomics allows the sequencing of the entire genome of the microorganisms present in a sample, such as the symptomatic or asymptomatic host plant, but also soil and other environmental matrices, constituting a valid approach for the identification of pathogens and therefore a precise diagnosis [93]. This technology has the considerable advantage of not requiring the previous isolation of pathogens in culture, which is impossible, for example, for the so-called obligate pathogens, and does not require specific probes or primers for each pathogen and therefore is not subjected to the biases that are often linked to amplification associated with PCR and metabarcoding. A flaw is represented by the rather complex analysis required by the data derived from sequencing: in fact, after the quality control of the reads and the assembly in contigs of the metagenome, these are subjected to “binning”, to form the complete genomes. Binning, through use of specific software, such as BUSCO [94] and CheckM [95], allows to assemble complete genomes and therefore to identify new pathogens, but it is almost difficult in samples with fragmented genomes, probably due to low coverage or in the case of very heterogeneous communities in which related species are present [96]. If a high sequencing depth is used, shotgun metagenomics allows for a precise taxonomic identification down to species level and, at least, for the most abundant species and for which the complete genomes are deposited in the databases. Recently, successful examples of species-level resolution by using HTS technologies were reported in literature: Yang and colleagues [97], by sequencing with the Oxford Nanopore Technologies MinION, distinguished the boxwood blight fungal pathogen *Calonectria pseudonaviculata* from *Calonectria henricotiae.* Loit et al. [98] compared the performances of two third-generation sequencing instruments, MinION (Oxford Nanopore Technologies) and Sequel (Pacific Biosciences), in identification and diagnostics of fungal and oomycete pathogens from *Pinaceae* and *Solanum* tissues by a metagenomic approach.

It is noteworthy to underline the importance of integrating metagenomics with metatranscriptomics to elucidate both the genomic potential and the species that are metabolically active in the total microbiome [99,100,101]. HTS has got a fundamental role in metatranscriptomics, allowing the whole transcriptome sequencing to detect isoforms, novel transcripts, alternative splice variants, and, as consequence, genomic variants. Garalde et al. [102] used Oxford Nanopore Technologies for directly sequencing of native RNA, bypassing reverse transcription and amplification and allowing to obtain full-length RNA sequences. Expressed genes can be efficiently annotated because of lacking introns, but as for metagenomics, the availability of complete genomes is of fundamental importance for taxonomic classification of fungal transcripts up to species level. Recently, Chialva et al. [103] used an RNA-seq dataset previously generated for tomato to detect the taxonomic and functional diversity of the root microbiota.

As already reported, the amount of sequence data derived from metagenomic and metatranscriptomic analysis is very large; it requires the availability of pipelines and user-friendly computational interfaces together with good knowledge of bioinformatics for its use, and this represents the main challenge in the use of metagenomics for routine diagnostics of plant pathogens. To support the analysis of HTS metadata from metagenomics and metatranscriptomics, several global-scale public databases, such as *MGnify* [104], *JGI/IMG* [105], and *MG-RAST* [106] combined with the *National Center for Biotechnology Information* (*NCBI*) *Taxonomy resource* [107], represent the most utilized ones. Very recently, a web resource named PREGO has been developed [108] with the aim of associating the processes occurring in one environment with the microorganisms involved in them.

## 5. Multilocus Analysis in Plant pathology: From Traditional to New-Generation Sequencing Technology

The target sequencing of multiple genomic loci (multilocus sequence typing—MLST) is considered one of the most reliable and informative methods for molecular genotyping [109]. It has been widely used to assess the extent of the genetic and pathogenic variability of different populations of the same genus (for example, *Colletotrichum* spp. [110]) and for taxonomic designation. In addition to ITS barcoding, MLST has become the standard method of genotyping of many fungi in studies of molecular epidemiology, pathogenicity, and phylogenetics. It is an important tool in describing and defining new species [111] in different fields, such as for fungal species of human concern, such as *Candida* [112], *Aspergillus* [113], and *Pseudallescheria* [114], or in plant pathology, i.e., *Colletotrichum* [115], *Ilyonectria* [116], or *Diaporthe* [117].

Conventional MLST involves the extraction of genomic DNA and the amplification by PCR of different conserved marker gene sequences, and in some cases, as few as three loci are sufficient to delineate species and strains. Moreover, online MLST databases are available for several bacterial and fungal species to support molecular epidemiological studies and surveillance. However, the current conventional MLST methodology has the disadvantages of being time consuming and costly due to the use of Sanger sequencing [109].

In a study on walnut anthracnose [118], multilocus analysis was performed by a combined approach for identifying *Colletotrichum* species. Both “traditional” Sanger identification, which involves the isolation and cultivation of pure isolates, and metabarcoding with ITS (Illumina MiSeq PE300) were used. It was thus possible to determine the species involved, with a good correspondence between the two methods although the limits of the metabarcoding approach emerged due to the occurrence of false negative and positive, to the low resolution of ITS in *Colletotrichum,* and to the plant DNA amplification using universal ITS1xITS4 primers (low depth analysis). However, in other cases, the metabarcoding approach could represent a more reliable method, especially when species-level marker genes are identified or when the method is based on a pair of primers specifically designed, as reported for *Fusarium* spp. by Cobo-Diaz et al. [39].

During last years, efforts have been made with the aim of resolving and overcoming the MLST disadvantages, especially for shortening the average time required for the analysis. For example, a high-throughput genotyping method (HiMLST) was developed by Boers et al. for typing four different bacterial species using 454 pyrophosphate sequencing [119]. Furthermore, a new high-throughput method of next-generation NGMLST genotyping has been developed for *Cryptococcus neoformans*/*Cryptococcus gatii* species complex [109], targeting the nine MLST loci commonly used to genotype those species complex isolates by a multiplexing protocol. The NGMLST method generates PacBio circular consensus sequencing (CCS) reads, which were automatically analyzed by a novel multifunctional software program, MLSTEZ. Chen et al. [109] compared those two NGS methods (HiMLST and NGMLST) with the conventional MLST based on 96 isolates with 8 target loci: the major advantages of NGMLST arise in terms of costs, time saving, and labor amount (Table 2). The application of NGMLST for *C. neoformans* and *C. gatii* has the potential to be used for any MLST study, as recently tested in different fields of application, such as the frequency estimation of multiple strains in one sample and their MLST type determination in human pathology for *Candida albicans* [120].

## 6. Phylogenomics

The interest in the sequencing of whole fungal genomes is acknowledged for many sectors, spanning industries including human and animal medicine, ecology, taxonomy, and agriculture [121]. As compared to previous phylogenetic studies that relied on few loci conserved among similar taxa, the comparison of whole fungal genomes is now providing the chance to better define a genome-scale fungal tree of life and the evolutionary processes at the base of fungal speciation in the light of characterization and preservation of fungal biodiversity related to the ecosystem services [122,123,124,125,126,127]. In agriculture, the knowledge of both plant and pathogen genomes is of outmost importance in defining the complex interactions that lead to the disease or to the resistance outcomes as well as the definition of sensible fungal targets for the development of new chemicals or natural products for their control. In molecular diagnostic of many phytopathogenic species, the availability of complete genome sequences offers an unpredictable tool for the definition of species boundaries [127], oomycetes hybrids identification [128], the evolution of virulence effectors [129], the identification of pathogens of quarantine relevance [130], and the characterization of genus of industrial relevance [131]. The future availability of even more whole fungal genomic data will boost research applications of both metabarcoding and metagenomic analyses to decipher the intricate interaction mechanisms between fungi and the surrounding environment.

The pipeline GEDI was developed by Feau et al. [132] to compare whole genomes of plant pathogens and phylogenetically related taxa by identifying genome regions specific and unique to targeted taxa or groups of related taxa for highly specific PCR assays. Since the genes targeted are unique, primer design is less constrained than with barcodes, and the likelihood of cross-interaction amongst the amplicons is reduced. This approach is promising to overcome some of the limitations of DNA detection assays based on conserved barcode genes. In their work, the authors applied the GEDI pipeline to some of the most important plant pathogens across three broad taxonomic groups: Phytophthora (*Stramenopiles*, *Oomycota*), *Dothideomycetes* (Fungi, Ascomycota), and *Pucciniales* (Fungi, *Basidiomycota*).

Feau et al. [133] also applied GEDI approach to develop a set of TaqMan real-time PCR detection assays targeting the DNA of all four *Phytophthora ramorum* lineages and the closely related species *P. lateralis.* The four lineages of *P. ramorum* (NA1, NA2, EU1, and EU2) differ in mating types and aggressiveness, yet share identical ITS sequences [134,135], so it is impossible for them to be differentiated with classical methods. Currently, the discrimination of the *P. ramorum* lineages is based on the use of time-consuming protocols. By using GEDI, Feau et al. [133] were able to identify unique loci in the four *P. ramorum* lineages that allowed to set up four qPCR assays (two primers and one probe each) specific for each lineage.

Pathogenic genetic determinants, which are putative specific markers in diagnosis, can also be identified by GWAS. In plant pathology, GWAS combines whole-genome sequencing and statistical methods to detect genomic regions associated with natural variation of disease resistance in plants and pathogenicity in pathogens [136]. The basic principle of GWAS is to screen one or multiple populations for genotypic and phenotypic differences between individuals [137]. GWAS can consider copy-number variants or sequence variations in the genomes although the most commonly studied genetic variants in GWAS are single-nucleotide polymorphisms (SNPs). With the rapid expansion of NGS technologies, the GWAS-based strategy allows to assess genetic variation at thousands of markers across the genomes, including those of non-model organisms, in order to find the variants statistically associated to a disease [138,139]. For these reasons, GWAS are being expected to open new roads in the field of phytopathology [140].

There are different examples of GWAS application to study variation of disease resistance in plants, but only few studies [141] employed GWA mapping to identify candidate pathogenic genetic determinants in bacterial, e.g., *Pseudomanas syringae* [142], and fungal pathogens, namely *Heterobasidium annosum* [143], *Fusarium graminearum* [144], *Parastagonospora nodorum* [145], *Puccinia triticina* [146], and *Colletotrichum kahawae* [147]. To our knowledge, no GWAS was reported yet on a phytopathogenic oomycete. The reasons for this paucity might be different; mainly, it is due to the use of comparative genomics as an efficient tool to identify important pathogenic genetic determinants though GWAS has the potential to analyze many individuals of several populations at the same time. Finally, understanding the number of mutational events and the potential of genetic variation of pathogen populations is essential in breeding for disease resistance.

## 7. Splitting of the Species *Pyrenochaeta lycopersici*

The availability of genomes from several “biotypes” of the same species often allows a more in-depth analysis for an accurate definition of fungal species at the whole-genome level. The identification of the accurate taxonomic level is essential for the diagnosis [148,149] both for the official control activities (monitoring the geographical distribution and/or border controls) and for the biological and functional information of the organism that derive from them (i.e., risk pest analysis). Despite the number of fungal genomes sequenced, assembled, and deposited in it, GenBank has already exceeded 10,000 [150], and the sequencing cost of an average fungal genome is drastically decreased, and the use of whole genomes in the definition of species is still reported in few published papers [151]. This is essentially due to the lack of tools and guidelines for data analysis. In 2019, Matute and Sepulveda attempted to outline standards for using genome sequences to delimit species boundaries [152] based on the following criteria: (1) monophyly, the proportion of loci within the genome that are mutually monophyletic is related to the time elapsed since species divergence; (2) concordance among genomic partitions as the use of thousands of markers distributed throughout the genome can allow to infer species tree based on a genome-wide concordance; (3) low shared polymorphism: the maintenance of ancestral polymorphism and gene exchange between divergent species are less likely as genetic divergences accumulate; and (4) lower interspecific differentiation compared to the intraspecific one: the average distance between individuals of two different species should be greater than the average distance between individuals within each of the species. Successively, other authors proposed several metrics to species delineation based on genomics data: pairwise comparison of assembled fungal genomes to calculate the genomic distance and determine species similarity thresholds, with over 90% accuracy [153]. In yeasts, similarly to prokaryote species delineation, the pairwise average nucleotide identity (ANI) values of 95% emerged as a good guideline for the species delineation [154]. All these proposed approaches must be considered with respect to the relative phylogenetic background of each taxon: for example, intraspecific distances must be compared with interspecific ones. Here, we examine a case study involving the comparison of genomic data of two “biotypes” formerly belonging to the species *Pyrenochaeta lycopersici*, a hemibiotrophic fungus of the class of Dothideomycetes, the agent of corky root rot (CRR), which is pathogenic for tomato and other Solanaceous species [155]. Previous analyses showed the existence of two biotypes of *P. lycopersici* on the basis of mycelium morphology, length of conidia, ITS sequence, and population analysis by random amplified polymorphic DNA (RAPD) and amplified fragment length polymorphism (AFLP) [156,157,158]. Despite morphological similarity, the ITS sequences showed only 89–90% of similarity, and population studies revealed a genetic differentiation between the two biotypes. To shed light on these ambiguous points, the genome of both the biotypes was sequenced and analyzed [159,160]. First, the genome of biotype 2 was sequenced using paired-end Illumina short-read technology that, despite having allowed to obtain new data to understand and encode the functions and the biology of this fungus, did not allow to represent the repeated sequence fraction of the genome in the final assembly. Successively, the genome sequencing of biotype 1 was performed using PacBio RS long-read technology, allowing to obtain a gapless genome assembly. Comparison between the two *P. lycopersici* isolates showed a genome size increase in type 1 (12.8%), most likely due to the resolution of longer repeats. However, in the aligned regions (over 97% of their length), there was a low sequence identity (mean 87.5%), and the number of genes of type 1 was slightly higher than that of type 2. Gene annotations of *P. lycopersici* type1 and type 2 were performed by the same software version to obtain comparable data. In 2017, Valenzuela-Lopez and coworkers [161] classified *P. lycopersici* in a new family: *Pseudopyrenochaetaceae*. They performed multi-locus sequence analysis of 357 strains belonging to different families within *Pleosporineae* and morphological study of 143 strains, classifying the two *P. lycopersici* “biotypes” as two different species, namely *Pseudopyrenochaeta lycopersici* and *Pseudopyrenochaeta terrestris*, confirming what emerged by genome analysis.

## 8. NGS as Resource in Certification and Quarantine Control Challenges

Description of new fungal pest species by NGS technologies represents an improved tool for biosecurity, regulatory, and commercial impact. The even more frequent discoveries of uncharacterized pathogens make it difficult to predict the impact of these new species on quarantine regulations and to manage the quarantine and certification pathogen lists [162]. If, on the one hand, the HTS technology has accelerated the discovery of new plant pests for which challenging decisions must be taken by National Plant Protection Organizations (NPPOs), on the other hand, information on biology and epidemiology as well as on pest risk analysis is limited and is acquired more slowly. For plant viruses, frameworks to manage this gap have been proposed to characterize these newly discovered pests and to promptly take appropriate action considering the right purposes [163]: this framework should be applied and formalized for any novel organism detected by HTS.

In 2018, Olmos et al. [164] described well the possibilities and opportunities of the use of HTS in routine plant pest diagnosis, listing four major applications: (i) surveillance programs, (ii) certifying plant propagation material, (iii) quarantine testing at borders, and (iv) monitoring of imported plant material for new potential risks. Subsequently, in 2019, the International Plant Protection Convention (IPPC) published a “recommendation on preparing the use of HTS as a diagnostic tool for phytosanitary purposes” to establish a guideline for harmonization, standardization, validation, and quality assurance applied to HTS tools [165]. Currently, metabarcoding and shotgun metagenomics are the most used HTS approaches in plant pest diagnosis and surveillance and are to be chosen according to the purpose and field of application (considering the answers to be obtained: “who is there?” and/or “what are they doing?”). The metabarcoding study gives information about the organisms present in the sample at a certain taxonomic level for fungi and other plant pests, and the standardized barcodes are proposed in a EPPO (European Plant Protection Organization) standard PM7/129 [166]. Targeted sequencing is limited to the analysis of taxa for those genome markers known taxonomically and reported in the available database [167]. Despite the limitations reported for the targeted sequencing in routine plant pest diagnostics, currently, metabarcoding sequencing is the most used approach for plant pest detection and identification due to the cost-efficiency ratio, the many well-developed tools available (including bioinformatics tools), and low false-positives risk. This last advantage represents a critical point especially for the official diagnosis of quarantine pest since the underlying economic implications.

The adoption of NGS analysis as a routine tool for the control of regulated plant pests involves the evaluation of some critical parameters, such as sensitivity, specificity, repeatability, and reproducibility. Unfortunately, there are still few peer-reviewed publications on the use of NGS for phytosanitary certification to be accepted by NPPOs, EPPO, or IPPC. While several works were made for pathogenic viruses and viroids [168,169], no studies concerning validation of HTS methods for diagnosis of phytopathogenic fungi have reported. The harmonization of diagnostic methods represents a critical point of plant protection policy, and this could be very difficult for the NGS technologies: the validation of these new diagnostic tests requires new experimental procedures and bioinformatics skills not foreseen in conventional routine analyses, which are constantly evolving and transforming. Currently, many phytosanitary services responsible for official diagnoses are accredited with ISO 17025, which, at the European level, has been implemented through the EPPO guidelines [170]. Even if the validation of HTS analysis procedures must meet the classic validation criteria defined in the IPPC and EPPO standards, more specific guidelines are needed for the quality assurance of the HTS approach. For example, the analytical sensitivity in HTS may depend on the number of reads generated per sample, on the DNA extraction method, in the case of amplicon sequencing on the competition for the primers in the PCR reaction, or differences in copy number of the targeted region between target organisms. It should be established using reference samples by comparing the results of dilution series of target samples with other reference tests [171]. Moreover, the analytical sensitivity value may be validated by defining the appropriate parameters in bioinformatic analysis. The analytical specificity of an HTS test depends on the sequencing strategy used, the genetic variability of the fungal target, the software and parameters used in the bioinformatic analyses and the reference sequence database, and, of course, on the desired taxonomic resolution. As for analytical sensitivity, the specificity of a metagenomics sequencing may depend on the number of sequenced reads and the appropriate target coverage, and a reproducible threshold level should be defined through reference target and non-target samples, validating the parameters by bioinformatic analysis.

## 9. Conclusions

It is now widely acknowledged that HTS technologies are of essential utility for diagnosis of filamentous plant pathogens, including Fungi and Oomycetes, and for improving plant disease management. However, there are still both economic and technical aspects to be considered before the dream comes true and is applicable at a broad-ranging level. The sequencing cost per sample is still not affordable for many laboratories even though this obstacle can be overcome by loading more samples in each run. This implies the need of additional steps of DNA purification to avoid unexpected and undesirable sequencing artifacts, such as the use of a mock sample and of DNA from healthy plant tissues as controls. When dealing with sequencing of soil samples, it has been often documented that sequences without any homologies in several DBs exist, i.e., the so-called “dark taxa” or “dark matter fungi” [172]. This has been ascribed to the huge amount of non-cultivable and undescribed fungal taxa but also to the low taxonomic resolution power using the “short-reads” sequences of the rRNA barcodes [173]. Long-read sequencing of the entire rRNA operon (including LSU, ITS, and SSU) has been proposed to partially solve this problem [15,174,175]. The proposal to allow intracellular DNA (metagenomic DNA or mgDNA) as types [176,177] has not been widely accepted by the scientific community [178]. Another pitfall in the use of NGS technology for routine fungal pathogen identification remains the need of improvement in processing the huge amount of NGS data both at infrastructure level (server and memory power) and bioinformatic skills availability (algorithms and expert technicians). To this aim, an ad hoc pipeline using machine learning (ML) classifiers has been developed as an alternative method for assigning individual error-prone sequence-long reads to taxa [28,179,180]. In metabarcoding studies, including those concerning human diseases, ML modeling will help in prediction of disease outputs and in deciphering environmental factors shaping the microbial composition also in agriculture and in natural ecosystems [29,181,182,183,184].

In conclusion, the high specialization of HTS technologies and the statistic management of data should not be a bottleneck for their adoption for diagnosis in plant pathology, providing that expertise in each field will be positively shared among researchers in a holistic approach [185,186]. We show here that NGS methodologies are now a reality for diagnosis and fungal identification when several and unknown pathogens are involved, such as for complex diseases or where environmental surveillance is needed. The availability of more and more complete fungal genomes in public databases will contribute to the spread of HTS technologies use for accurate diagnosis of fungal plant pathogens and for discovering novel pathogens, especially those considered unculturable.

## Figures and Tables

**Table 1:** Step procedures in metabarcoding approach (discussed in the text).

Sampling
Optimization of sample collection and extraction of samples free of contamination
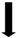
DNA extraction
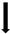
Amplicon production
Choice of suitable primers for barcode genes (Custom/Universal) PCR amplification/library preparation
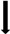
High-Throughput DNA Sequencing (HTS)
Different sequencing platforms Short reads/Long reads
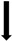
Sequencing data analysis
Sequence Clustering (OTU/ASV) Taxonomic/Functional assignments (Comparison to Databases) (Analyzed with different bioinformatic pipelines)

**Table 2:** Comparison between NGMLST, HiMLST, and conventional MLST based on 96 isolates with 8 target loci (adapted from [109]).

	NGMLST	HiMLST	Conventional MLST
PCR amplifications	192	864	768
PCR product purifications	4	>96	768
Estimated time for experimental work	7 h	>30 h	>1 week
Estimated time for data analysis	≈1 h	>10 h	>10 h
Data analysis	automatic (by specific software)	manual	manual
Estimated cost per isolate	EUR 9.6	EUR 46	EUR 77
